# Causal role of immune cells in hypertension: a bidirectional Mendelian randomization study

**DOI:** 10.3389/fcvm.2024.1375704

**Published:** 2024-05-27

**Authors:** Xinhe Zhang, Guanying Li, Wei Wu, Bin Li

**Affiliations:** ^1^ Department of Cardiology, Central Hospital Affiliated to Shandong First Medical University, Jinan, China; ^2^ Shandong First Medical University, Jinan, China; ^3^ Research Center of Translational Medicine, Central Hospital Affiliated to Shandong First Medical University, Jinan, China; ^4^ Jinan Foreign Language School International Center, Jinan, China; ^5^ Department of Cardiology, Hekou District People Hospital, Dongying, China

**Keywords:** Mendelian randomization, immune cell, hypertension, prehypertension, two-sample mendelian randomization

## Abstract

**Background:**

Although Hypertension (HTN) is considered to be a cardiovascular disease caused by multiple factors, the cause of it is still unknown. In this study, we aim to find out whether circulating immune cell characteristics have an impact on susceptibility to HTN.

**Methods:**

This study employed a comprehensive two-sample Mendelian randomization (MR) analysis to investigate the causal association between immune cell characteristics and HTN. Utilizing publicly accessible genetic data, we examined the causal relationship between HTN and the susceptibility to 731 immune cell signatures. To ensure the reliability and validity of the findings, a comprehensive sensitivity analysis was conducted to assess heterogeneity, confirm the robustness of the results and evaluate the presence of horizontal pleiotropy.

**Results:**

After FDR correction, immune phenotype had an effect on HTN. In our study, one immunophenotype was identified as being positively associated with HTN risk significance: HLA DR on CD33- HLA DR+. In addition, we examined 8 immune phenotype with no statistically significant effect of HTN, but it is worth mentioning that they had an unadjusted low *P*-value phenotype.

**Conclusions:**

Our MR study by genetic means demonstrated the close relationship between HTN and immune cells, thus providing guidance for future clinical prediction and subsequent treatment of HTN.

## Introduction

HTN, as well as prehypertension and other dangerous forms of HTN, is responsible for 85 million deaths worldwide from stroke, ischaemic heart disease, other vascular diseases and kidney disease (1). Between 1990 and 2019, the number of people with high blood pressure worldwide has absolutely doubled in the age group 30–79, and to this day, high blood pressure remains the leading cause of death and disability worldwide, far outweighing the influence of other occupational, environmental and lifestyle factors. Despite the availability of treatments, global blood pressure control rates are only 18%–23%, and as low as 10% in some parts of Asia and Africa. Although the low blood pressure control rates are partly due to poor treatment adherence, they also highlight that the cause of elevated blood pressure in most adults with HTN is unknown (2). However, there is no systematic study on the causal relationship between various immune cell signatures and HTN.

Cardiovascular and cerebrovascular diseases are mostly diseases with poor prognosis and relatively sudden onset, such as stroke, coronary artery disease, atrial fibrillation and peripheral vascular diseases, and HTN happens to be their main pathogenic factor (3). Recently, with the aging of the population and the acceleration of social development, the proportion of people suffering from HTN in the total population is increasing year by year and the age of disease is gradually getting younger. Moreover, because of its high prevalence, HTN is the single largest risk factor for heart failure in the population (4). And in the early stages, HTN may not be detected because it is usually asymptomatic (5). The World Health Organization (WHO) estimates that about 46% of people with HTN in the world are unaware of their condition. Therefore, there is an urgent need for an indicator that can prompt early HTN or even prehypertension.

MR was originally developed as an alternative to randomised controlled trials (RCTS) to provide reliable causal evidence between exposure and outcome through genetic variation (6). It has since evolved into an independent analytical method based on the principles of Mendelian distribution, primarily used for epidemiological etiological inference. The validity of the causal sequence in MR is of utmost importance (7, 8). It serves as the basis for natural experiments through the random allocation of alleles during meiosis cell division and conception (6, 9). Currently, MR has been widely applied in disease research, but there are no relevant reports to elucidate the causal relationship between immune cells and HTN.

In this study, a comprehensive two-sample MR Analysis was performed to determine the causal relationship between immune cell characteristics and HTN, which will provide new insights into the prevention and treatment of HTN in the future.

## Materials and methods

### Study design

Based on a two-sample MR analysis, we evaluated the causal relationship between 731 immune cell features (7 groups) and HTN. MR uses genetic variation to represent risk factors, therefore, effective instrumental variables in causal inference must satisfy three key assumptions (10):

1. Association hypothesis: SNPS are strongly correlated with exposure factors.
2. Independence hypothesis: SNPS are independent of confounders.
3. Exclusivity hypothesis: SNPS can only have an effect on outcomes through exposure.

The study we analyzed received approval from the relevant institutional review committee, and participants provided informed consent forms (11, 12).

### Genome-wide association study (GWAS) data sources for HTN

We from Integrative Epidemiology Unit (IEU) open GWAS HTN analysis of the database access to data, the database is mainly composed of a publicly available GWAS summary data sets. We used the HTN GWAS Summary statistics from FinnGen. The GWAS consisted of 55,917 cases and 162,837 controls. This MR Study was conducted using GWAS aggregate statistics and received ethical approval from each GWAS.

### Immunity-wide GWAS data sources

The GWAS catalog provides publicly accessible GWAS summary statistics for immune coverage, specifically for each immune trait (GCST0001391 to GCST0002121) (13). This study successfully identified 122 significant independent association signals at 70 locations, with 53 being previously unreported. Additionally, it elucidated the molecules and mechanisms responsible for regulating 459 cellular features related to immune function. A comprehensive analysis was conducted using flow cytometry to examine a total of 731 immunophenotypes, encompassing absolute cell counts (*n* = 118), median fluorescence intensity (MFI) as an indicator of surface antigen levels (*n* = 389), morphological parameters (MP) (*n* = 32), and relative cell counts (*n* = 192).The MFI, AC, and RC features encompass B cells, CDC, mature stage T cells, monocytes, bone marrow cells, TBNK (T cells, B cells, natural killer cells) and Treg panels, whereas the MP features consist of CDC and TBNK panels. The initial immunological profile Genome-Wide Association Study (GWAS) was carried out utilizing data from 3,757 individuals of European descent, with no cohorts overlapping. Approximately 22 million single nucleotide polymorphisms (SNPs) were genotyped using high-density arrays, utilizing a reference panel derived from Sardinian sequences. The genotyping of the samples was conducted using four Illumina arrays, namely OHTNiExpress, ImmunoChip, Cardio-MetaboChip, and ExomeChip. Subsequently, associations were examined while accounting for covariates such as sex, age (14).

### Selection of instrumental variables (IVs)

According to recent studies (14, 15), the significance level of the IV for each immunological trait was set to 1 × 10^−5^ In order to ensure that SNP effects on each immune trait and HTN are associated with the same allele, the direction of effects needs to be coordinated. Furthermore, SNPs exhibiting linkage imbalance (with an r2 threshold of <0.001 within a 10 Mb window) were excluded from the obtained dataset, while the remaining SNPs were retained. To ensure the robustness of the exposure, any statistic with a small *f*-statistic (*F* < 10) was also eliminated from our analyses. We did the same thing with the metabolite data. Finally, we also identified 9 IVs of HTN for further reverse MR Analysis.

### Statistical analysis

All analyses were performed in R 3.5.3 software (http://www.Rproject.org). This study mainly used the software package “MendelianRandomization” (version 0.4) (16) to evaluate the causal relationship between 731 immunophenotypes and HTN. It is used to perform inverse variance weighting (IVW) (17), weighted median based methods (18), and model-based methods (19). The Cochran's *Q* statistic and its associated *p*-values are employed to examine the presence of heterogeneity among the chosen independent variables (IVs). In the event that the null hypothesis is rejected, a random effect inverse variance weighting (IVW) is utilized in lieu of a fixed effect IVW (17).We used a common method, namely MR-Egger, to exclude the effect of horizontal pleiotropy. If the intercept term is significant, it indicates the presence of horizontal multiplicity. Additionally, we employed a powerful method called MR-PRESSO to exclude potential horizontal pleiotropic outliers that could significantly impact the estimated results in the MR-PRESSO package (19). Furthermore, scatter plots, funnel plots and MR leave-one-out sensitivity analysis plots are employed to analyze the data. The scatter plot provides evidence that the outcomes remain unaltered by outliers, while the funnel plot serves to illustrate the strength of the correlation and the absence of heterogeneity. The “MR leave-one-out sensitivity analysis” method refers to the gradual elimination of each SNP, the calculation of the meta effect of the remaining SNP, and the observation of whether the result changes after the elimination of each SNP. If the result changes greatly after the elimination of a SNP, it indicates that there is a SNP that has a great impact on the result, which is used to verify the sensitivity analysis. Consequently, the scatter plot reaffirms the resilience of the results against outliers.

## Results

### Some immune cells influence susceptibility to HTN

**Figure 1:**
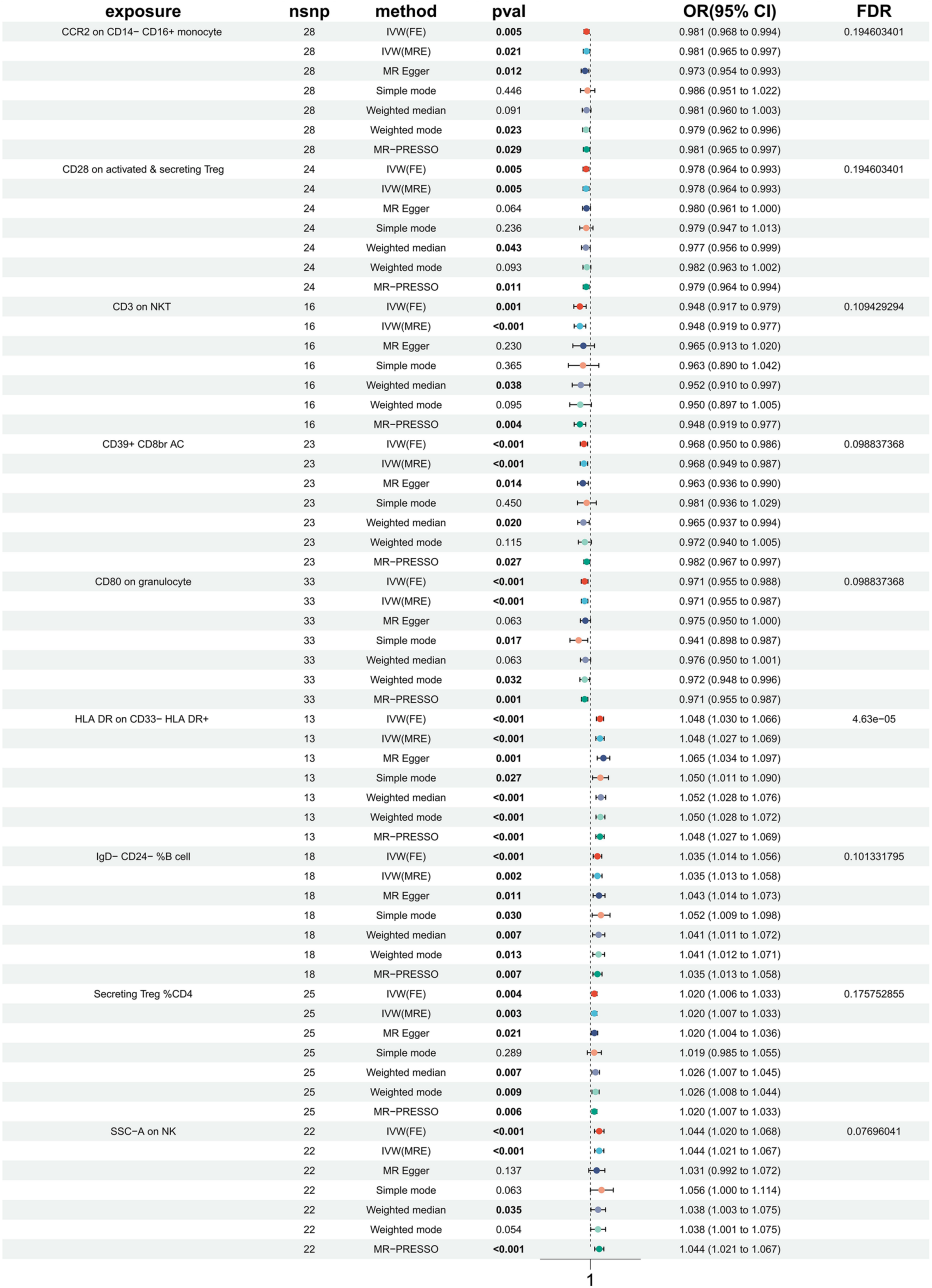
Forest plots showed the causal associations between immune cell and HTN.

In order to investigate the causal impact of immunophenotypes on HTN, a two-sample MR analysis was conducted. The inverse-variance weighted (IVW) analysis was considered the most effective approach, provided that valid instrumental variables (IVs) were utilized. Additionally, when the genetic IVs exhibited no pleiotropic effects and the sample size was sufficiently large, the IVW estimate demonstrated consistency, efficiency, and proximity to the true value (20). Hence, the IVW method was selected as the primary approach for conducting MR analysis in order to investigate the causal impact of HTN on immunophenotype. Two samples were utilized for this analysis, with a particular focus on the IVW method. At a nominal significance level, we identified causal relationships between HTN and 63 immune cells. Specifically, elevated levels of 31 immune cells and reduced levels of 32 immune cells were found to be associated with an increased risk of HTN. Following multiple test adjustments using the false discovery rate (FDR) method (PFDR < 0.05), we observed that one immunophenotype exhibited a significant association with HTN risk: We observed a significant causal effect of HLA DR on CD33- HLA DR+ on HTN risk by using the Inverse variance weighted (fixed effects) (OR = 1.048, CI: 1.030–1.066, *P* = 6.34 × 10^−8^, PFDR = 4.63 × 10^−5^, Figure 1, Supplementary Table S1, Tables 1, 2), which is consistent with weighted mode (*P* = 0.0007), weighted median (*P* = 0.000014), MR-PRESSO (*P* = 0.00062), Simple mode (*P* = 0.027), MR Egger (*P* = 0.0015). It is also noteworthy that eight suggestive immunophenotypes were identified at a significance of 0.20. Four of them, respectively CD28 on activated & secreting Treg (OR = 0.978, CI: 0.964–0.993, *P* = 0.005, PFDR = 0.195, Figure 1, Supplementary Table S1, Tables 1, 2), CCR2 on CD14- CD16+ monocyte (OR = 0.981, CI: 0.968–0.994, *P* = 0.005, *PFDR* = 0.195, Figure 1, Supplementary Table S1, Tables 1, 2), CD3 on NKT (OR = 0.948, CI: 0.917–0.979, *P* = 0.001, PFDR = 0.109, Figure 1, Supplementary Table S1, Tables 1, 2), CD80 on granulocyteT (OR = 0.971, CI: 0.955–0.988, *P* = 0.0008, PFDR = 0.099, Figure 1, Supplementary Table S1, Tables 1, 2) and CD39+ CD8br AC (OR = 0.968, CI: 0.950–0.986, *P* = 0.0006, PFDR = 0.099, Figure 1, Supplementary Table S1, Tables 1, 2) have a negative effect on the incidence of HTN, whereas the remaining three, IgD- CD24- %B cell (OR = 1.035, CI: 1.014–1.056, *P* = 0.00097, PFDR = 0.101, Figure 1, Supplementary Table S1, Tables 1, 2), Secreting Treg %CD4 (OR = 1.020, CI: 1.006–1.033, *P* = 0.004, PFDR = 0.18, Figure 1, Supplementary Table S1, Tables 1, 2) and SSC-A (OR = 1.044, CI: 1.020–1.068, *P* = 0.0002, PFDR = 0.077, Figure 1, Supplementary Table S1, Tables 1, 2) on NK, have a positive effect on the incidence of HTN. Using the other methods and sensitivity analysis, we confirmed the robustness of the causal associations observed.

**Table 1:** Causal effects of immune cells on HTN.

Id.outcome	Outcome	Exposure	Method	nsnp	b	se	pval	lo_ci	up_ci	or	or_lci95	or_uci95	fdr
finn-b-I9_HYPTENS	Coronary heart disease	CD28 on activated & secreting Treg	Inverse variance weighted (fixed effects)	24	−0.021928662	0.007734499	0.004580054	−0.03708828	−0.006769043	0.978310023	0.963591065	0.993253815	0.194603401
finn-b-I9_HYPTENS	Coronary heart disease	CD3 on NKT	Inverse variance weighted (fixed effects)	16	−0.053694561	0.016575185	0.001197585	−0.086181923	−0.021207199	0.947721534	0.917427315	0.979016092	0.109429294
finn-b-I9_HYPTENS	Coronary heart disease	CD39+ CD8br AC	Inverse variance weighted (fixed effects)	23	−0.032807336	0.009595241	0.000628227	−0.051614007	−0.014000664	0.967724988	0.949695371	0.98609689	0.098837368
finn-b-I9_HYPTENS	Coronary heart disease	CD80 on granulocyte	Inverse variance weighted (fixed effects)	33	−0.029239351	0.00873096	0.000811251	−0.046352033	−0.012126669	0.971183983	0.954705815	0.987946562	0.098837368
finn-b-I9_HYPTENS	Coronary heart disease	Secreting Treg %CD4	Inverse variance weighted (fixed effects)	25	0.019512307	0.006684162	0.003509475	0.00641135	0.032613265	1.019703917	1.006431947	1.033150906	0.175752855
finn-b-I9_HYPTENS	Coronary heart disease	IgD- CD24- %B cell	Inverse variance weighted (fixed effects)	18	0.034458887	0.010445295	0.000970346	0.013986109	0.054931664	1.035059473	1.014084372	1.056468418	0.101331795
finn-b-I9_HYPTENS	Coronary heart disease	HLA DR on CD33- HLA DR+	Inverse variance weighted (fixed effects)	13	0.046655385	0.008625523	6.34E-08	0.02974936	0.063561411	1.047760873	1.030196293	1.065624924	4.63E-05
finn-b-I9_HYPTENS	Coronary heart disease	CCR2 on CD14- CD16+ monocyte	Inverse variance weighted (fixed effects)	28	−0.019294614	0.00684036	0.004791876	−0.03270172	−0.005887509	0.980890335	0.9678272	0.994129789	0.194603401
finn-b-I9_HYPTENS	Coronary heart disease	SSC-A on NK	Inverse variance weighted (fixed effects)	22	0.042978637	0.011597056	0.000210562	0.020248408	0.065708866	1.043915593	1.020454797	1.067915765	0.07696041

**Table 2:** Causal effects of immune cells on HTN.

											Heterogeneity test					Pleiotropy test			MR-PRESSO global test		
Id.outcome	Outcome	Exposure	Method	nsnp	b	se	pval	lo_ci	up_ci	or	or_lci95	or_uci95	FDR	Q	Q_df	Q_pval	egger _intercept	se	pval	RSSobs	*P* value
finn-b-I9_HYPTENS	Coronary heart disease	HLA DR on CD33- HLA DR+	IVW(FE)	13	0.046655385	0.008625523	6.34E-08	0.02974936	0.063561411	1.047760873	1.030196293	1.065624924	4.63E-05	16.62990202	12	0.164057408	−0.009368797	0.006582628	0.182398926	18.94929976	0.255
finn-b-I9_HYPTENS	Coronary heart disease	HLA DR on CD33- HLA DR+	IVW(MRE)	13	0.046655385	0.010154059	4.33E-06	0.02675343	0.06655734	1.047760873	1.027114516	1.068822249									
finn-b-I9_HYPTENS	Coronary heart disease	HLA DR on CD33- HLA DR+	MR Egger	13	0.062811925	0.014961584	0.001490065	0.03348722	0.092136631	1.064826553	1.034054228	1.096514629		14.04372265	11	0.230583561					
finn-b-I9_HYPTENS	Coronary heart disease	HLA DR on CD33- HLA DR+	Simple mode	13	0.048615034	0.019287338	0.02688118	0.010811851	0.086418218	1.049816129	1.01087051	1.0902622									
finn-b-I9_HYPTENS	Coronary heart disease	HLA DR on CD33- HLA DR+	Weighted median	13	0.050460982	0.011631221	1.44E-05	0.027663789	0.073258174	1.051755825	1.028049985	1.076008298									
finn-b-I9_HYPTENS	Coronary heart disease	HLA DR on CD33- HLA DR+	Weighted mode	13	0.048615034	0.010767916	0.000708172	0.027509919	0.069720149	1.049816129	1.027891811	1.072208081									
finn-b-I9_HYPTENS	Coronary heart disease	HLA DR on CD33- HLA DR+	MR-PRESSO	13	0.046655385	0.010154059	0.000616498	0.02675343	0.06655734	1.047760873	1.027114516	1.068822249									
finn-b-I9_HYPTENS	Coronary heart disease	CD3 on NKT	IVW(FE)	16	−0.053694561	0.016575185	0.001197585	−0.086181923	−0.021207199	0.947721534	0.917427315	0.979016092	0.109429294	13.15161789	15	0.590588214	−0.005307609	0.006808689	0.448642947	14.58839816	0.659
finn-b-I9_HYPTENS	Coronary heart disease	CD3 on NKT	IVW(MRE)	16	−0.053694561	0.015520379	0.000540945	−0.084114505	−0.023274617	0.947721534	0.919325983	0.976994148									
finn-b-I9_HYPTENS	Coronary heart disease	CD3 on NKT	MR Egger	16	−0.03567582	0.028443418	0.230280716	−0.09142492	0.02007328	0.964953061	0.912629834	1.020276103		12.54394374	14	0.562705427					
finn-b-I9_HYPTENS	Coronary heart disease	CD3 on NKT	Simple mode	16	−0.037454119	0.040077976	0.364831383	−0.116006951	0.041098713	0.963238611	0.890469034	1.041954955									
finn-b-I9_HYPTENS	Coronary heart disease	CD3 on NKT	Weighted median	16	−0.048779119	0.023450926	0.037520869	−0.094742934	−0.002815304	0.952391472	0.909606734	0.997188656									
finn-b-I9_HYPTENS	Coronary heart disease	CD3 on NKT	Weighted mode	16	−0.05169241	0.028998921	0.094904028	−0.108530295	0.005145475	0.949620915	0.897151715	1.005158735									
finn-b-I9_HYPTENS	Coronary heart disease	CD3 on NKT	MR-PRESSO	16	−0.053694561	0.015520379	0.003502045	−0.084114505	−0.023274617	0.947721534	0.919325983	0.976994148									
finn-b-I9_HYPTENS	Coronary heart disease	CD28 on activated & secreting Treg	IVW(FE)	24	−0.021928662	0.007734499	0.004580054	−0.03708828	−0.006769043	0.978310023	0.963591065	0.993253815	0.194603401	23.23452419	23	0.447147607	−0.001508178	0.004268336	0.727196271	24.73997432	0.502
finn-b-I9_HYPTENS	Coronary heart disease	CD28 on activated & secreting Treg	IVW(MRE)	24	−0.021928662	0.007773833	0.004789954	−0.037165374	−0.00669195	0.978310023	0.963516782	0.993330391									
finn-b-I9_HYPTENS	Coronary heart disease	CD28 on activated & secreting Treg	MR Egger	24	−0.019719933	0.01009444	0.063587963	−0.039505035	6.52E-05	0.980473233	0.961265114	1.000065172		23.10341255	22	0.395910001					
finn-b-I9_HYPTENS	Coronary heart disease	CD28 on activated & secreting Treg	Simple mode	24	−0.020943926	0.017200696	0.235713301	−0.05465729	0.012769439	0.979273875	0.946809573	1.012851316									
finn-b-I9_HYPTENS	Coronary heart disease	CD28 on activated & secreting Treg	Weighted median	24	−0.022881184	0.011305248	0.04297595	−0.04503947	−0.000722899	0.977378605	0.955959749	0.999277362									
finn-b-I9_HYPTENS	Coronary heart disease	CD28 on activated & secreting Treg	Weighted mode	24	−0.017730227	0.010121515	0.093144539	−0.037568398	0.002107943	0.982426028	0.96312854	1.002110166									
finn-b-I9_HYPTENS	Coronary heart disease	CD28 on activated & secreting Treg	MR-PRESSO	24	−0.021227559	0.007649765	0.010523559	−0.036221099	−0.00623402	0.978996159	0.964427036	0.993785372									
finn-b-I9_HYPTENS	Coronary heart disease	CD39+ CD8br AC	IVW(FE)	23	−0.032807336	0.009595241	0.000628227	−0.051614007	−0.014000664	0.967724988	0.949695371	0.98609689	0.098837368	23.20947194	22	0.389983286	0.002718217	0.005118156	0.600927043	25.73913095	0.39
finn-b-I9_HYPTENS	Coronary heart disease	CD39+ CD8br AC	IVW(MRE)	23	−0.032807336	0.009855466	0.000872064	−0.052124049	−0.013490622	0.967724988	0.949211111	0.986599969									
finn-b-I9_HYPTENS	Coronary heart disease	CD39+ CD8br AC	MR Egger	23	−0.038136961	0.014181401	0.013733927	−0.065932506	−0.010341416	0.962581096	0.93619405	0.989711873		22.9018674	21	0.349232911					
finn-b-I9_HYPTENS	Coronary heart disease	CD39+ CD8br AC	Simple mode	23	−0.018783294	0.02440793	0.449747013	−0.066622836	0.029056249	0.981392013	0.935547989	1.0294825									
finn-b-I9_HYPTENS	Coronary heart disease	CD39+ CD8br AC	Weighted median	23	−0.035431655	0.015208555	0.019821024	−0.065240423	−0.005622887	0.965188698	0.936842198	0.994392892									
finn-b-I9_HYPTENS	Coronary heart disease	CD39+ CD8br AC	Weighted mode	23	−0.02812455	0.017128007	0.114804527	−0.061695444	0.005446344	0.972267263	0.940169177	1.005461202									
finn-b-I9_HYPTENS	Coronary heart disease	CD39+ CD8br AC	MR-PRESSO	23	−0.018120399	0.007679443	0.027157562	−0.033172107	−0.003068691	0.982042789	0.967372054	0.996936013									
finn-b-I9_HYPTENS	Coronary heart disease	CD80 on granulocyte	IVW(FE)	33	−0.029239351	0.00873096	0.000811251	−0.046352033	−0.012126669	0.971183983	0.954705815	0.987946562	0.098837368	29.46649328	32	0.595392904	−0.001474275	0.003668079	0.69050052	31.42404849	0.657
finn-b-I9_HYPTENS	Coronary heart disease	CD80 on granulocyte	IVW(MRE)	33	−0.029239351	0.00837821	0.00048315	−0.045660643	−0.012818059	0.971183983	0.955366118	0.987263742									
finn-b-I9_HYPTENS	Coronary heart disease	CD80 on granulocyte	MR Egger	33	−0.025298378	0.013129158	0.063203156	−0.051031529	0.000434772	0.975018944	0.95024871	1.000434867		29.30495349	31	0.55335449					
finn-b-I9_HYPTENS	Coronary heart disease	CD80 on granulocyte	Simple mode	33	−0.060342813	0.023998653	0.017145471	−0.107380172	−0.013305453	0.94144174	0.898184143	0.986782673									
finn-b-I9_HYPTENS	Coronary heart disease	CD80 on granulocyte	Weighted median	33	−0.024761972	0.013305369	0.062736955	−0.050840495	0.001316551	0.975542091	0.950430257	1.001317418									
finn-b-I9_HYPTENS	Coronary heart disease	CD80 on granulocyte	Weighted mode	33	−0.028715474	0.012819873	0.032170944	−0.053842426	−0.003588522	0.971692897	0.947581409	0.996417909									
finn-b-I9_HYPTENS	Coronary heart disease	CD80 on granulocyte	MR-PRESSO	33	−0.029239351	0.00837821	0.001431154	−0.045660643	−0.012818059	0.971183983	0.955366118	0.987263742									
finn-b-I9_HYPTENS	Coronary heart disease	CCR2 on CD14- CD16+ monocyte	IVW(FE)	28	−0.019294614	0.00684036	0.004791876	−0.03270172	−0.005887509	0.980890335	0.9678272	0.994129789	0.194603401	40.25713983	27	0.048466598	0.007022944	0.005172313	0.186196698	42.29640187	0.083
finn-b-I9_HYPTENS	Coronary heart disease	CCR2 on CD14- CD16+ monocyte	IVW(MRE)	28	−0.019294614	0.008352539	0.02088651	−0.035665592	−0.002923637	0.980890335	0.964962931	0.997080633									
finn-b-I9_HYPTENS	Coronary heart disease	CCR2 on CD14- CD16+ monocyte	MR Egger	28	−0.027109589	0.010038849	0.012017527	−0.046785734	−0.007433445	0.973254578	0.954291848	0.992594115		37.59159378	26	0.066050579					
finn-b-I9_HYPTENS	Coronary heart disease	CCR2 on CD14- CD16+ monocyte	Simple mode	28	−0.014224511	0.018400961	0.446219303	−0.050290394	0.021841373	0.98587618	0.950953233	1.022081642									
finn-b-I9_HYPTENS	Coronary heart disease	CCR2 on CD14- CD16+ monocyte	Weighted median	28	−0.019097872	0.01128376	0.090548863	−0.041214042	0.003018298	0.981083337	0.959623708	1.003022858									
finn-b-I9_HYPTENS	Coronary heart disease	CCR2 on CD14- CD16+ monocyte	Weighted mode	28	−0.021481974	0.008882508	0.022603623	−0.03889169	−0.004072257	0.978747121	0.961854882	0.995936023									
finn-b-I9_HYPTENS	Coronary heart disease	CCR2 on CD14- CD16+ monocyte	MR-PRESSO	28	−0.019294614	0.008352539	0.028766688	−0.035665592	−0.002923637	0.980890335	0.964962931	0.997080633									
finn-b-I9_HYPTENS	Coronary heart disease	IgD- CD24- %B cell	IVW(FE)	18	0.034458887	0.010445295	0.000970346	0.013986109	0.054931664	1.035059473	1.014084372	1.056468418	0.101331795	19.58454922	17	0.296028263	−0.004666021	0.005672027	0.422805659	20.95987609	0.389
finn-b-I9_HYPTENS	Coronary heart disease	IgD- CD24- %B cell	IVW(MRE)	18	0.034458887	0.011211224	0.002114884	0.012484888	0.056432885	1.035059473	1.01256315	1.058055601									
finn-b-I9_HYPTENS	Coronary heart disease	IgD- CD24- %B cell	MR Egger	18	0.041894633	0.014485508	0.010611901	0.013503038	0.070286228	1.042784598	1.013594616	1.072815207		18.78981961	16	0.279713014					
finn-b-I9_HYPTENS	Coronary heart disease	IgD- CD24- %B cell	Simple mode	18	0.051060839	0.021527374	0.029768284	0.008867186	0.093254492	1.052386917	1.008906616	1.097741066									
finn-b-I9_HYPTENS	Coronary heart disease	IgD- CD24- %B cell	Weighted median	18	0.040144604	0.014914123	0.007108517	0.010912922	0.069376285	1.04096129	1.010972685	1.07183945									
finn-b-I9_HYPTENS	Coronary heart disease	IgD- CD24- %B cell	Weighted mode	18	0.040033497	0.014420571	0.012938514	0.011769177	0.068297817	1.040845639	1.011838706	1.070684129									
finn-b-I9_HYPTENS	Coronary heart disease	IgD- CD24- %B cell	MR-PRESSO	18	0.034458887	0.011211224	0.006882773	0.012484888	0.056432885	1.035059473	1.01256315	1.058055601									
finn-b-I9_HYPTENS	Coronary heart disease	Secreting Treg %CD4	IVW(FE)	25	0.019512307	0.006684162	0.003509475	0.00641135	0.032613265	1.019703917	1.006431947	1.033150906	0.175752855	22.48474082	24	0.550358506	−0.000255556	0.003445731	0.941519558	24.6227533	0.568
finn-b-I9_HYPTENS	Coronary heart disease	Secreting Treg %CD4	IVW(MRE)	25	0.019512307	0.006469717	0.002561801	0.006831662	0.032192953	1.019703917	1.006855051	1.032716752									
finn-b-I9_HYPTENS	Coronary heart disease	Secreting Treg %CD4	MR Egger	25	0.019839506	0.00800882	0.021023121	0.00414222	0.035536793	1.020037617	1.004150811	1.036175771		22.47924022	23	0.491500143					
finn-b-I9_HYPTENS	Coronary heart disease	Secreting Treg %CD4	Simple mode	25	0.018961434	0.017496218	0.289245833	−0.015331154	0.053254021	1.019142343	0.98478577	1.054697527									
finn-b-I9_HYPTENS	Coronary heart disease	Secreting Treg %CD4	Weighted median	25	0.025526701	0.009439317	0.006844942	0.00702564	0.044027762	1.025855297	1.007050377	1.045011366									
finn-b-I9_HYPTENS	Coronary heart disease	Secreting Treg %CD4	Weighted mode	25	0.025299355	0.008922952	0.009145688	0.007810369	0.042788341	1.0256221	1.007840949	1.04371696									
finn-b-I9_HYPTENS	Coronary heart disease	Secreting Treg %CD4	MR-PRESSO	25	0.019512307	0.006469717	0.005974852	0.006831662	0.032192953	1.019703917	1.006855051	1.032716752									
finn-b-I9_HYPTENS	Coronary heart disease	SSC-A on NK	IVW(FE)	22	0.042978637	0.011597056	0.000210562	0.020248408	0.065708866	1.043915593	1.020454797	1.067915765	0.07696041	19.32848056	21	0.564077291	0.003572481	0.004679927	0.454158325	20.82943873	0.618
finn-b-I9_HYPTENS	Coronary heart disease	SSC-A on NK	IVW(MRE)	22	0.042978637	0.011125946	0.00011204	0.021171782	0.064785491	1.043915593	1.021397495	1.066930134									
finn-b-I9_HYPTENS	Coronary heart disease	SSC-A on NK	MR Egger	22	0.030718779	0.019809745	0.136656321	−0.00810832	0.069545879	1.03119547	0.991924464	1.072021243		18.74575809	20	0.538402987					
finn-b-I9_HYPTENS	Coronary heart disease	SSC-A on NK	Simple mode	22	0.054043212	0.027571193	0.063383113	3.67E-06	0.10808275	1.055530213	1.000003674	1.114139937									
finn-b-I9_HYPTENS	Coronary heart disease	SSC-A on NK	Weighted median	22	0.03760945	0.017868043	0.035304854	0.002588086	0.072630813	1.038325635	1.002591438	1.075333465									
finn-b-I9_HYPTENS	Coronary heart disease	SSC-A on NK	Weighted mode	22	0.037077626	0.018155124	0.053882366	0.001493584	0.072661668	1.037773576	1.0014947	1.075366644									
finn-b-I9_HYPTENS	Coronary heart disease	SSC-A on NK	MR-PRESSO	22	0.042978637	0.011125946	0.000901211	0.021171782	0.064785491	1.043915593	1.021397495	1.066930134									

**Figure 2:**
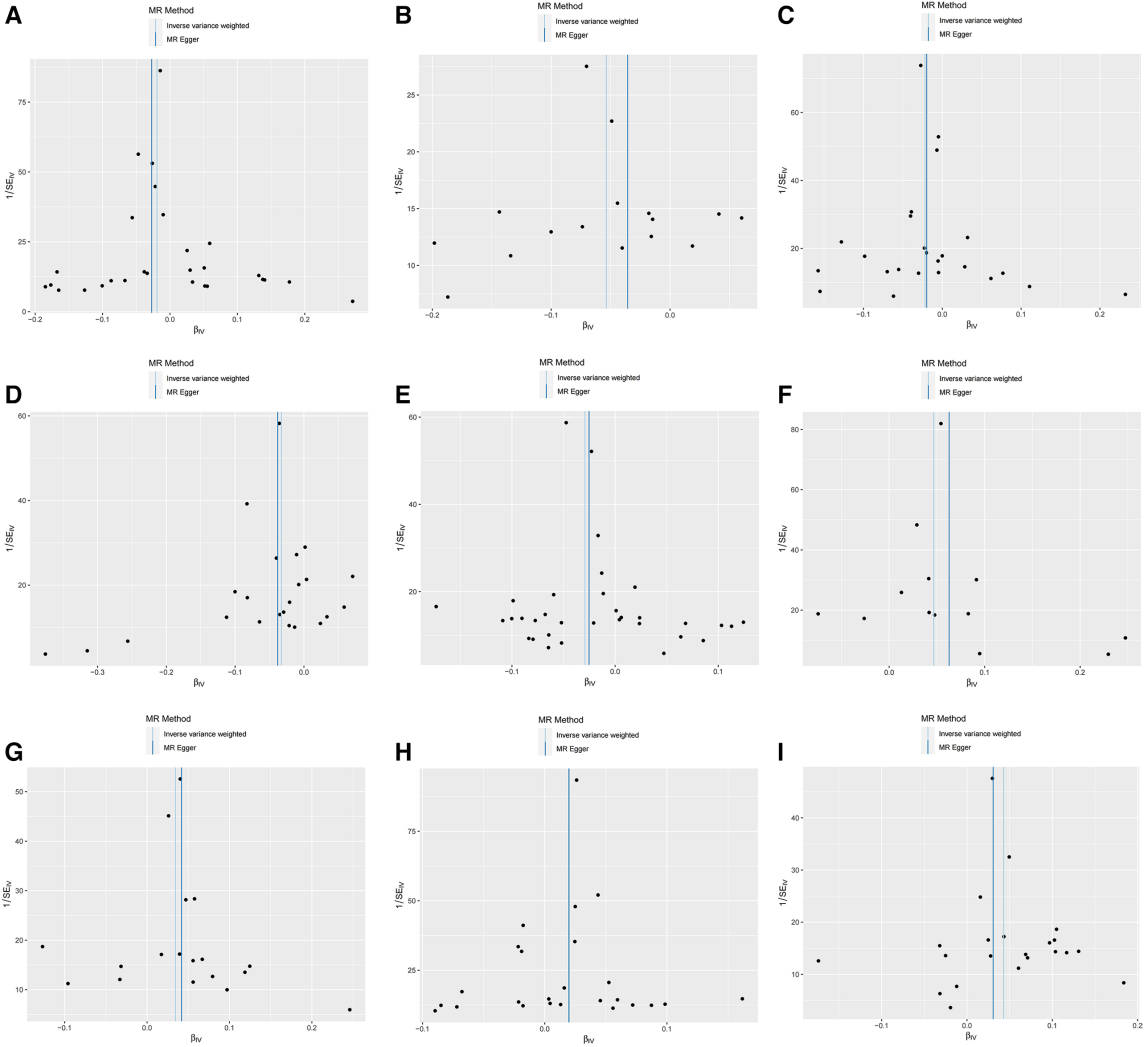
Scatter plots, funnel plots for casual associations between immune cell and HTN risk. (**A**) Funnel plots for CCR2 on CD14- CD16+ monocyte on hypertension. (**B**) Funnel plots for CD3 on NKT on hypertension. (**C**) Funnel plots for CD28 on activated & secreting Treg on hypertension. (**D**) Funnel plots for CD39+ CD8br AC on hypertension. (**E**) Funnel plots for CD80 on granulocyte on hypertension. (**F**) Funnel plots for HLA DR on CD33- HLA DR+ on hypertension. (**G**) Funnel plots for IgD- CD24- _B cell on hypertension. (**H**) Funnel plots for Secreting Treg _CD4 on hypertension. (**I**) Funnel plots for SSC-A on NKon hypertension. (**J**) Scatter plots for CCR2 on CD14- CD16+ monocyte on hypertension. (**K**) Scatter plots for CD3 on NKT on hypertension. (**L**) Scatter plots for CD28 on activated & secreting Treg on hypertension. (**M**) Scatter plots for CD39+ CD8br AC on hypertension. (**N**) Scatter plots for CD80 on granulocyte on hypertension. (**O**) Scatter plots for HLA DR on CD33- HLA DR+ on hypertension. (**P**) Scatter plots for IgD- CD24- _B cell on hypertension. (**Q**) Scatter plots for Secreting Treg _CD4 on hypertension. (**R**) Scatter plots for SSC-A on NK.scatter on hypertension.

**Figure 3:**
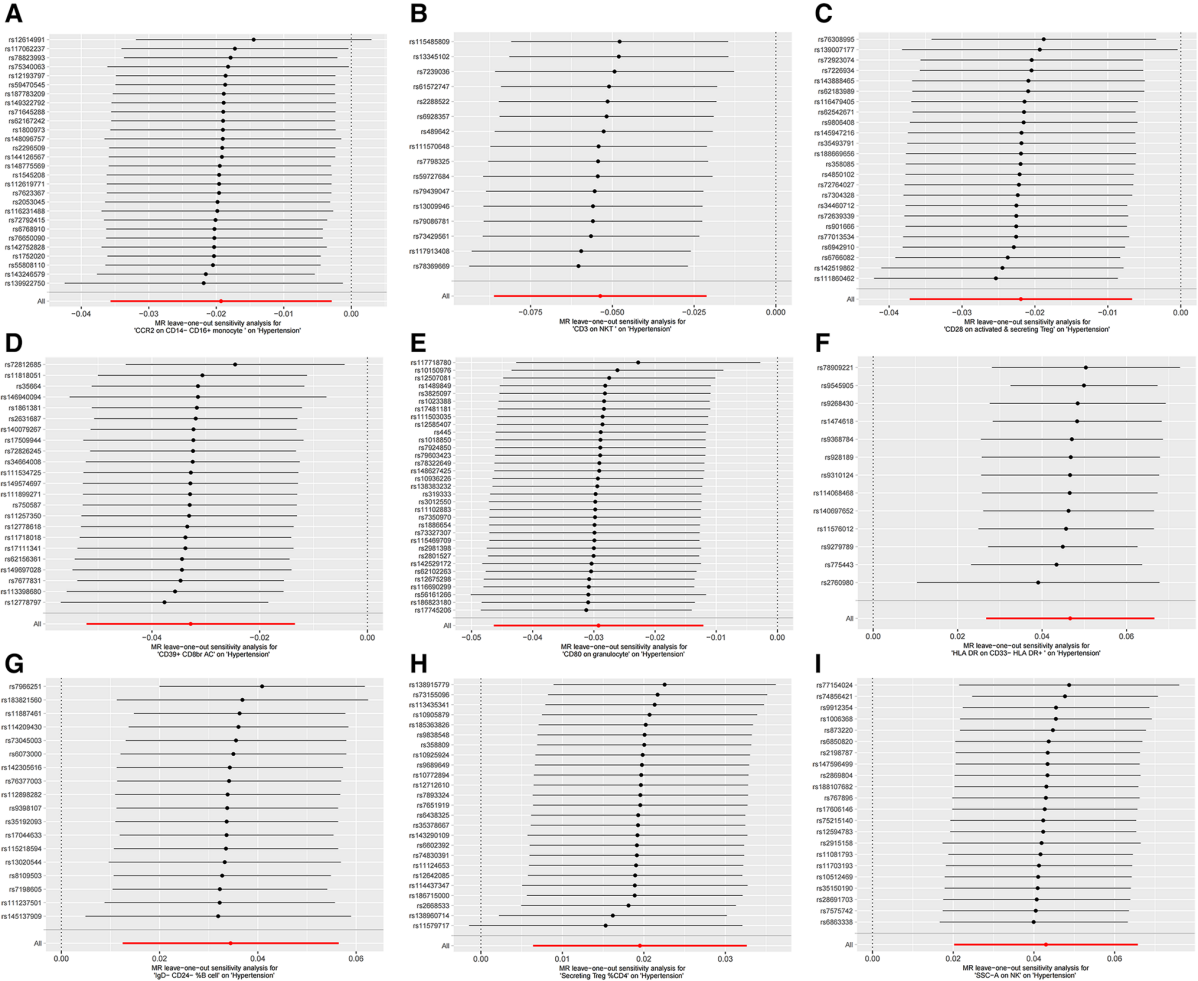
MR leave-one-out sensitivity analysis plots for casual associations between immune cell and HTN risk. (**A**) Leave-one-out plots for CCR2 on CD14- CD16+ monocyte on hypertension. (**B**) MR MR leave-one-out sensitivity analysis sensitivity analysis plots for CD3 on NKT on hypertension. (**C**) Leave-one-out plots for CD28 on activated & secreting Treg on hypertension. (**D**) MR leave-one-out sensitivity analysis plots for CD39+ CD8br AC on hypertension. (**E**) MR leave-one-out sensitivity analysis plots for CD80 on granulocyte on hypertension. (**F**) MR leave-one-out sensitivity analysis plots for HLA DR on CD33- HLA DR+ on hypertension. (**G**) MR leave-one-out sensitivity analysis plots for IgD- CD24- _B cell on hypertension. (**H**) MR leave-one-out sensitivity analysis plots for Secreting Treg _CD4 on hypertension. (**I**) MR leave-one-out sensitivity analysis plots for SSC-A on NK on hypertension.

Furthermore, the MR-Egger intercept and the global test of MR-PRESSO effectively eliminated the potential influence of horizontal pleiotropy (Supplementary Table S1). The stability of the findings was further supported by the scatter plots, funnel plots and MR leave-one-out sensitivity analysis plots (Figures 2, 3).

**Figure:**
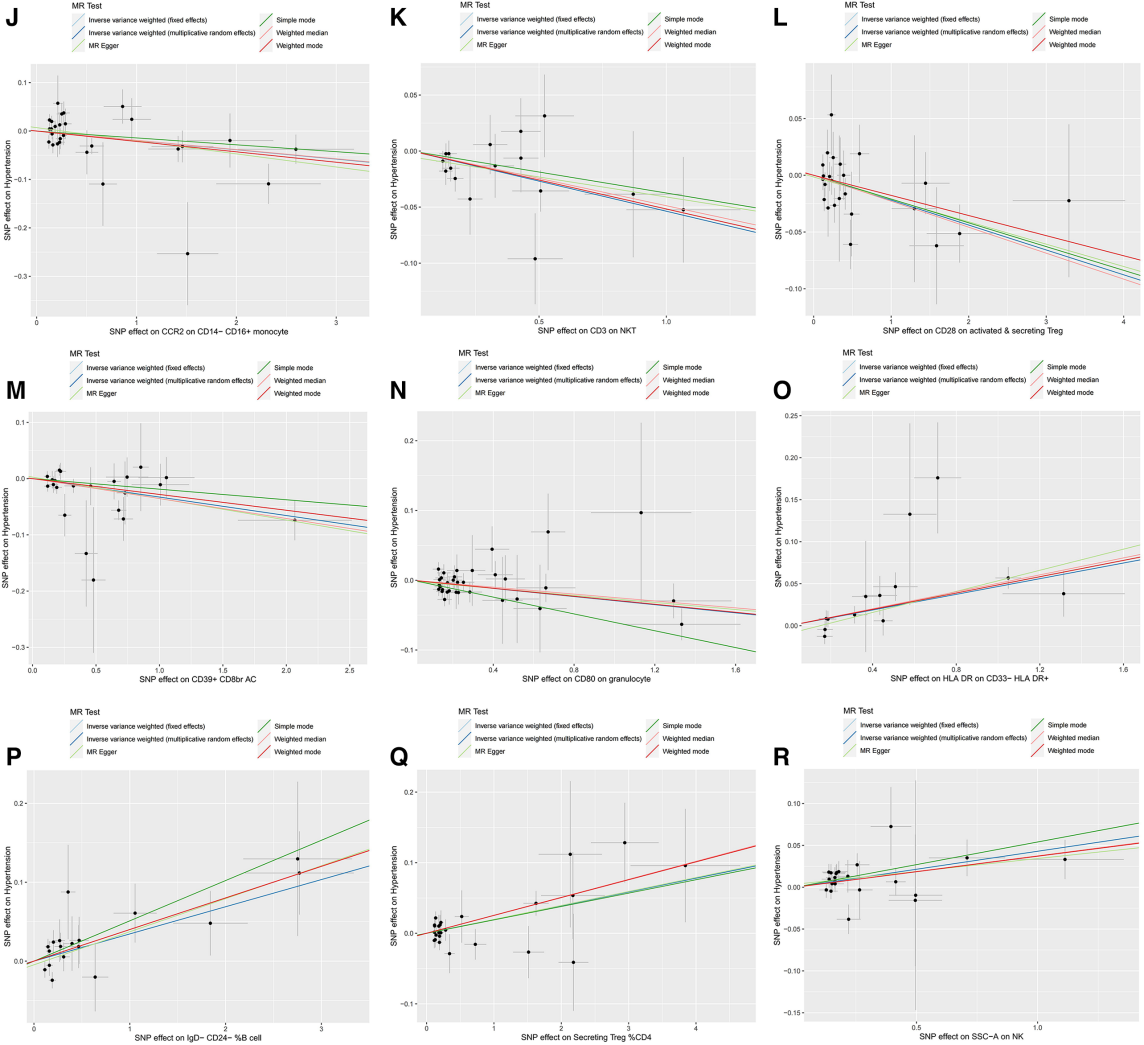


### Exploration of the causal effect of immunophenotypes on HTN

**Figure 4:**
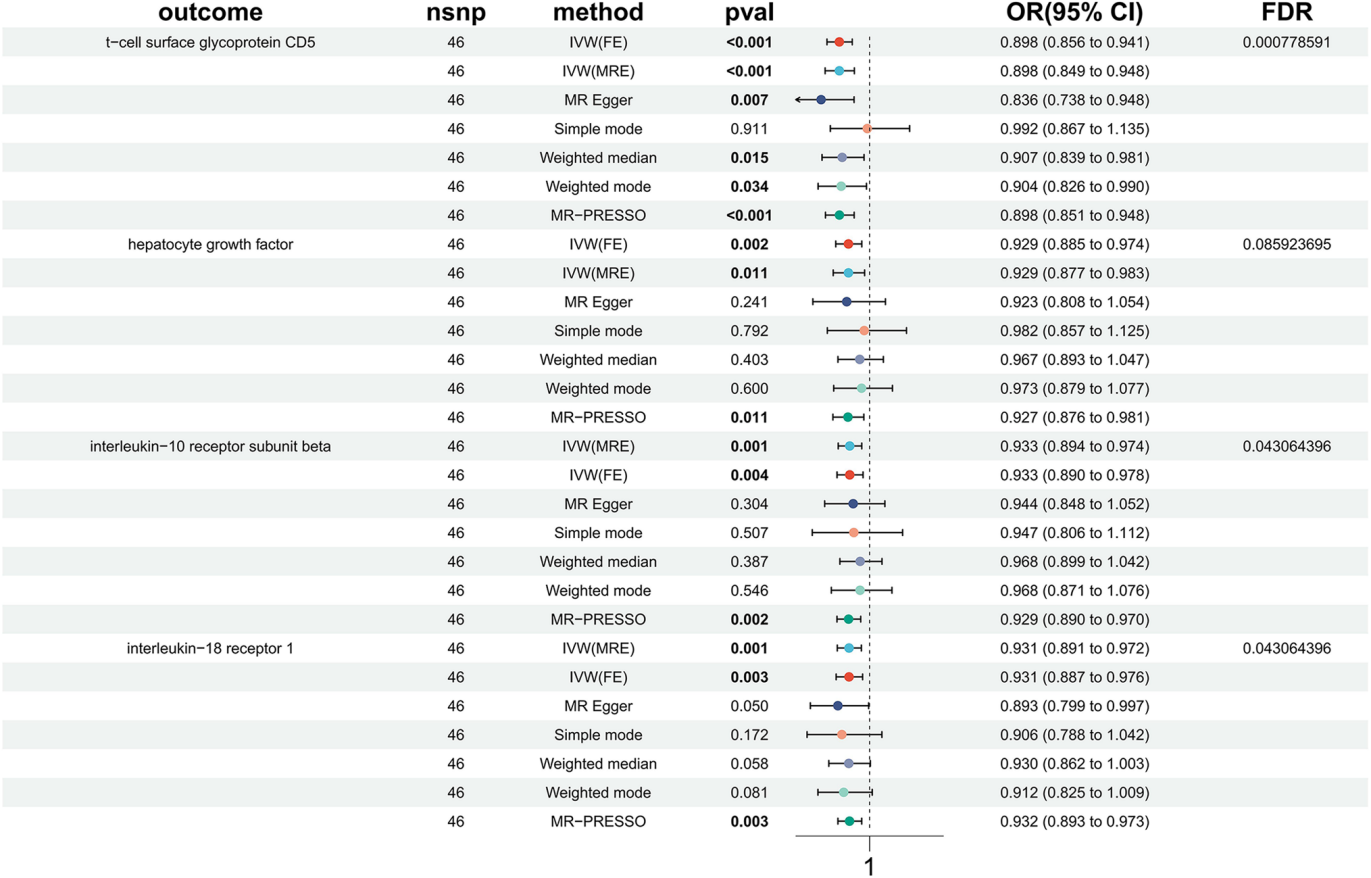
Forest plots of the causal associations between HTN and immune cell.

To investigate the impact of HTN on the body's immune system, we conducted an analysis using MR to explore the causal effects of HTN on immune cells. Despite applying multiple test adjustments, we did not observe a statistically significant causal relationship at a false discovery rate (FDR) significance level of 0.05. However, at the nominal significance level, we did detect causal effects of HTN on the levels of 15 immune cells. Specifically, the onset of HTN was found to increase the levels of 5 immune cells and decrease the levels of 10 immune cells. These 7 immune cells are distributed across B cells (15 cells), myeloid cells (3 cells), and TBNK cells (2 cells) (Figure 4, Table 3, Supplementary Table S2).

**Table 3:** Causal effects of immune cell on HTN.

Id.exposure	Id.outcome	Outcome	Exposure	Method	nsnp	b	se	pval	lo_ci	up_ci	or	or_lci95	or_uci95
finn-b-I9_HYPTENS	ebi-a-GCST90001526	CD33dim HLA DR+ CD11b+ %CD33dim HLA DR+ || id:ebi-a-GCST90001526	Hypertension	Inverse variance weighted (fixed effects)	56	−0.15948768	0.073596112	0.030229772	−0.30373606	−0.015239301	0.852580471	0.738055643	0.984876229
finn-b-I9_HYPTENS	ebi-a-GCST90001528	CD33dim HLA DR+ CD11b- %CD33dim HLA DR+ || id:ebi-a-GCST90001528	Hypertension	Inverse variance weighted (fixed effects)	56	0.162507934	0.073645265	0.027339841	0.018163215	0.306852654	1.176457653	1.018329169	1.35914069
finn-b-I9_HYPTENS	ebi-a-GCST90001597	CD8dim T cell %T cell || id:ebi-a-GCST90001597	Hypertension	Inverse variance weighted (fixed effects)	56	0.159121463	0.053318905	0.002841956	0.05461641	0.263626516	1.172480351	1.056135414	1.301641963
finn-b-I9_HYPTENS	ebi-a-GCST90001612	CD8dim T cell %leukocyte || id:ebi-a-GCST90001612	Hypertension	Inverse variance weighted (fixed effects)	56	0.154342036	0.053890684	0.004183496	0.048716296	0.259967776	1.166889936	1.049922441	1.296888295
finn-b-I9_HYPTENS	ebi-a-GCST90001634	CD8dim Natural Killer T %T cell || id:ebi-a-GCST90001634	Hypertension	Inverse variance weighted (fixed effects)	56	0.147563861	0.053677148	0.005975911	0.04235665	0.252771072	1.159007298	1.043266493	1.287588478
finn-b-I9_HYPTENS	ebi-a-GCST90001635	CD8dim Natural Killer T %lymphocyte || id:ebi-a-GCST90001635	Hypertension	Inverse variance weighted (fixed effects)	56	0.142508477	0.053439872	0.007659843	0.037766329	0.247250625	1.153162856	1.03848854	1.280499998
finn-b-I9_HYPTENS	ebi-a-GCST90001743	CD20 on B cell || id:ebi-a-GCST90001743	Hypertension	Inverse variance weighted (fixed effects)	55	−0.108838069	0.054097561	0.044232066	−0.214869288	−0.002806849	0.896875638	0.806646872	0.997197086
finn-b-I9_HYPTENS	ebi-a-GCST90001745	CD20 on CD24+ CD27+ B cell || id:ebi-a-GCST90001745	Hypertension	Inverse variance weighted (fixed effects)	56	−0.134964893	0.053329933	0.011381794	−0.239491563	−0.030438223	0.873746586	0.787027914	0.970020355
finn-b-I9_HYPTENS	ebi-a-GCST90001754	CD20 on IgD- CD27- B cell || id:ebi-a-GCST90001754	Hypertension	Inverse variance weighted (fixed effects)	49	−0.121053042	0.057737952	0.036029534	−0.234219429	−0.007886656	0.885986963	0.791188187	0.992144362
finn-b-I9_HYPTENS	ebi-a-GCST90001755	CD20 on IgD- CD38- B cell || id:ebi-a-GCST90001755	Hypertension	Inverse variance weighted (fixed effects)	56	−0.112337063	0.053460142	0.03561256	−0.217118942	−0.007555185	0.893742959	0.804834235	0.992473283
finn-b-I9_HYPTENS	ebi-a-GCST90001757	CD20 on IgD- CD38dim B cell || id:ebi-a-GCST90001757	Hypertension	Inverse variance weighted (fixed effects)	56	−0.163311415	0.053291431	0.002180366	−0.26776262	−0.05886021	0.849326654	0.765089377	0.942838559
finn-b-I9_HYPTENS	ebi-a-GCST90001758	CD20 on memory B cell || id:ebi-a-GCST90001758	Hypertension	Inverse variance weighted (fixed effects)	56	−0.148102642	0.05326061	0.005423913	−0.252493438	−0.043711846	0.862342598	0.776861311	0.957229747
finn-b-I9_HYPTENS	ebi-a-GCST90001761	CD20 on switched memory B cell || id:ebi-a-GCST90001761	Hypertension	Inverse variance weighted (fixed effects)	56	−0.153307154	0.053687111	0.004296003	−0.258533892	−0.048080417	0.857866184	0.77218286	0.953057142
finn-b-I9_HYPTENS	ebi-a-GCST90001803	CD27 on IgD- CD38+ B cell || id:ebi-a-GCST90001803	Hypertension	Inverse variance weighted (fixed effects)	53	−0.117906702	0.054800668	0.031432345	−0.225316011	−0.010497394	0.88877897	0.798263919	0.989557511
finn-b-I9_HYPTENS	ebi-a-GCST90002111	HLA DR on CD33dim HLA DR+ CD11b- || id:ebi-a-GCST90002111	Hypertension	Inverse variance weighted (fixed effects)	56	−0.174149797	0.077783009	0.025161237	−0.326604496	−0.021695099	0.840171033	0.721368991	0.978538547

## Discussion

HTN is the leading preventable cause of premature death worldwide (21, 22). HTN constitutes a significant risk factor for both ischemic and hemorrhagic stroke, as well as coronary artery disease. Furthermore, individuals affected by HTN are susceptible to the development of kidney failure, heart failure, peripheral vascular disease, and various other medical conditions (23). Over the past twelve years, the utilization of genome-wide association studies (GWAS) and whole exome sequencing (WES) techniques has led to the identification of numerous potential pathways associated with blood pressure. However, the occurrence of consistent associations with HTN remains relatively infrequent (24). These findings are not exclusive to HTN and have been observed in other intricate chronic conditions, including anemia and diabetes, involving pathway gene variants rather than consistent disease variants (25, 26). It is indisputable that numerous countries have advocated for and implemented measures to prioritize HTN as a health concern, exemplified by the 2020 US Surgeon General's directive to regulate HTN (27–29).

With so much attention around the world, and with so many clinical trials demonstrating the efficacy of different classes of antihypertensive drugs (30), HTN is still not completely under control despite improvements in treatment. From the perspective of preventive medicine, the new treatment strategy for HTN is to intervene when blood pressure is normally high, aiming to suppress or even reverse HTN, which is worth looking forward to (31). Providing early treatment in the prehypertension stage may cause the HTN to subside (32). Once HTN occurs, depending on the cause of the disease, intermittent intensive treatment can bring the HTN into remission. We believe that early HTN is a good indication for intermittent treatment. Biomarkers are urgently needed to accurately predict natural disease history and prognosis.

Our study employs a comprehensive integration of large-scale individual and aggregated Genome-Wide Association Study (GWAS) datasets to systematically elucidate the genetic mechanism underlying the immune cell response to the occurrence and progression of HTN. To the best of our knowledge, this is the inaugural MR analysis investigating the causal relationship between multiple immunophenotypes and HTN. Among the four immune features examined in this study, namely Mean Fluorescence Intensity (MFI), Receptor Count (RC), Antibody Count (AC), and Membrane Potential (MP), we identified one immunophenotype that exhibited a significant causal effect on HTN (FDR < 0.05). Additionally, it is worth noting that eight immunophenotypes demonstrated suggestive causal effects on HTN (FDR < 0.2). HTN had suggestive causal effects on 15 immunophenotypes (*P* < 0.05).

Based on our comprehensive data analysis and extensive research, we have determined that the presence of HLA DR on CD33- HLA DR+ significantly augments the susceptibility to HTN. It is noteworthy that HLA-DR, an MHC class II cell surface receptor, is encoded by the human leukocyte antigen complex situated on the 6P21 region of chromosome 6 (11). There is growing evidence that HTN occurs simultaneously with, and may be caused by, changes in complement, inflammasome activation, and circulating immune cell phenotypes, particularly bone marrow cells. These inflammatory processes are interconnected and ultimately contribute to the adaptive immune system by means of oxidative stress, endogenous protein modification, and perturbed antigen processing and presentation mechanisms (33). There is growing evidence that infiltration of bone marrow cells (especially monocytes/macrophages and T cells) into the arteries and kidneys is an early marker of inflammation (34, 35), and that these cells can produce various inflammatory cytokines that contribute to HTN (36–39). There is an increasing amount of evidence suggesting that HTN is associated with and possibly caused by the activation of complement, inflammasome, and changes in the phenotype of circulating immune cells, particularly bone marrow cells. These inflammatory processes are interconnected and ultimately contribute to the adaptive immune system through oxidative stress, modification of endogenous proteins, and disrupted mechanisms of antigen processing and presentation (40). Increased inflammation within the cardiac regulatory center of the brain is associated with heightened activation of the sympathetic nervous system, leading to elevated blood pressure. Consequently, mitigating this inflammatory response can potentially ameliorate HTN (41). In addition, recent studies have demonstrated that abnormal bone marrow cells can lead to pulmonary HTN (42).

## Conclusions

We conclude that immune cells are plausible causal agents of HTN based on our two-way MR analysis. These results offer valuable support to clinical decision-making regarding disease prognosis and treatment, while also guiding the exploration of novel therapeutic interventions. Moreover, they underscore the intricate nature of the interplay between the immune system and HTN. A further advantage of our study is that unavoidable confounding variables, reverse causation, and other relevant factors are effectively mitigated. Even so, it is clear that HTN is complex in its pathogenesis, and that immune cells associated with HTN exhibit clinical heterogeneity. Additionally, solitary treatments frequently fail to yield satisfactory outcomes. Consequently, further investigation is warranted to explore the interplay between innate immune cells and the interaction between innate immune cells and adaptive immune cells in individuals with HTN.

## Data Availability

The original contributions presented in the study are included in the article/**Supplementary Material**, further inquiries can be directed to the corresponding authors.
